# Persistent *C. pneumoniae* infection in atherosclerotic lesions: rethinking the clinical trials

**DOI:** 10.3389/fcimb.2014.00034

**Published:** 2014-03-21

**Authors:** Lee Ann Campbell, Michael E. Rosenfeld

**Affiliations:** ^1^Department of Epidemiology, University of WashingtonSeattle, WA, USA; ^2^Department of Environmental and Occupational Health Sciences, Department of Pathology, University of WashingtonSeattle, WA, USA

**Keywords:** *Chlamydia pneumoniae*, atherosclerosis, persistence, antibiotics, clinical trials

The hypothesis that infectious agents are a risk factor for atherosclerosis has implicated multiple viral and bacterial pathogens in contributing either directly or indirectly to disease progression (Rosenfeld and Campbell, 2011). One of the most vigorously studied organisms has been *Chlamydia pneumoniae*, which has been associated with cardiovascular disease by seroepidemiological studies, detection of the organism by multiple methods in atherosclerotic tissue, and experimental studies demonstrating biological plausibility. Significantly, *C. pneumoniae* accelerates lesion progression in mouse and rabbit models of atherosclerosis (Muhlestein et al., 1998; Hu et al., 1999; Moazed et al., 1999; Fong, 2000; Blessing et al., 2001). Early small clinical trials determined whether treatment with macrolides (Azithromycin, Roxithromycin, and Clarithromycin) would be efficacious in secondary prevention of coronary heart disease. These studies yielded mixed results and had several limitations including the small numbers of patients and short duration of treatment and follow-up period (Grayston, 2003). However, half of them demonstrated some beneficial effects, which provided enthusiasm for the potential of antibiotic intervention in coronary artery disease. There have since been four large clinical trials collectively enrolling over 20,000 patients with stable coronary artery disease (WIZARD, ACES, and CLARICOR) or acute coronary syndrome (PROVE-IT–TIMI) (O'Connor et al., 2003; Cannon et al., 2005; Grayston et al., 2005; Jespersen et al., 2006). As there were short term beneficial effects in the WIZARD trial following a 3 month course of Azithromycin, two subsequent studies addressed whether longer term treatment would be efficacious in reducing coronary events. In the ACES study, patients were treated with Azithromycin for 1 year and followed for 46.8 months (Grayston et al., 2005). The PROVE IT-TIMI trial treated with gatifloxacin for a mean duration of 2 years (Cannon et al., 2005). Overall, none of these well-designed trials demonstrated any long term benefit of antibiotic treatment. Furthermore, two large scale trials subsequently found that treatment with either roxithromycin or rifalizil (PROVIDENCE-1) had no beneficial effects in patients with peripheral artery disease (Joensen et al., 2008; Jaff et al., 2009). Cumulatively, these trials clearly demonstrated that anti-chlamydial antibiotics should not be recommended for treatment of patients with coronary heart disease or peripheral artery disease. Prior to the completion of the PROVE-IT and ACES trials, Grayston commented that if the trials demonstrated a beneficial effect of antibiotics, this would provide additional evidence for a role of *C. pneumoniae* in pathogenesis, but would not prove causality (Grayston, 2003). Alternatively, he predicted that negative results would mostly likely dampen interest in the association of *C. pneumoniae* and atherosclerosis, but noted that failure of the clinical trials would not rule out a pathogenic role (Grayston, 2003). Indeed, the negative outcome led some to conclude that this proved that *C. pneumoniae* did not play a role in the pathogenesis of atherosclerosis (Danesh, 2005) and diminished interest in infectious agents as contributing factors for cardiovascular disease (Epstein et al., 2009).

However, other investigators have underscored several factors that warrant critical evaluation before dismissing *C. pneumoniae* as a contributor to atherosclerotic processes (Anderson, 2005; Taylor-Robinson and Boman, 2005; Deniset and Pierce, 2010; Muhlestein, 2011; Rosenfeld and Campbell, 2011). First, treatment was given to patients with “end stage of disease” that is likely not modifiable. By analogy, antibiotic treatment is not effective in individuals in which inflammation resulting from chronic *C. trachomatis* infection of the upper genital tract or eye has led to the fibrosis and scarring observed in tubal factor infertility and trachoma, respectively. Whether antibiotics would be efficacious in treatment of patients with early atherosclerosis has not been determined as such studies would be difficult to design and execute. Second, it is possible that antibiotic treatment might be ineffective due to pathogen burden as viruses or other bacteria contributing to atherosclerotic processes may not be susceptible to the chosen antibiotics (Epstein et al., 2009; Rosenfeld and Campbell, 2011). Third, the patients in the large scale trials had advanced atherosclerosis and the events being measured were likely due to plaque destabilization and rupture, events that may be independent of plaque progression due to infection. Fourth, a single antibiotic was used in the trials and it is possible that treatment with a combination of antibiotics might be more effective as shown for patients with chronic *Chlamydia*-induced reactive arthritis (Carter et al., 2010). Last, and the focus of this opinion, is the ability of chlamydiae to establish persistent/chronic infection and the difficulty in treating such infections (Beatty et al., 1994; Grayston, 2003). Chlamydiae undergo a developmental cycle in which the elementary body, an infectious but metabolically inactive form, is not susceptible to antibiotics. The reticulate body, the intracellular replicating form, can establish persistence, a state in which the developmental cycle is arrested rendering the organism refractory to antibiotics. In a continuous cell culture model of *C. pneumoniae* infection thought to more accurately mimic *in vivo* conditions, a mixture of developmental forms, including aberrant forms characteristic of persistent organisms, are observed. In this model, prolonged treatment with antibiotics including azithromycin and clarithromycin failed to eliminate infection (Kutlin et al., 2002a,b). It was also demonstrated in infected monocytes *in vitro* and monocytes isolated from patients undergoing treatment with azithromycin for coronary artery disease that infection was recalcitrant to antibiotic treatment (Gieffers et al., 2001). In addition, various antibiotics can induce chlamydial persistence in cell culture, including azithromycin (Beatty et al., 1994; Gieffers et al., 2004; Wyrick and Knight, 2004). Recently, amoxicillin has been shown to result in the induction of reversible persistent *Chlamydia muridarum* infection in a mouse model of genital tract infection (Phillips Campbell, 2012). The ability of *C. pneumoniae* to establish persistent infection *in vivo* has been experimentally validated in a mouse model of lung infection (Malinverni et al., 1995a; Laitinen et al., 1996). At times post-infection in which the organism can no longer be cultured from the lungs (but pathology persists and the organism can be detected by PCR), treatment with cortisone acetate results in reactivation of infection and the ability to culture organisms. Importantly, in animal models, the organism is frequently detected by PCR or immunohistochemistry following treatment with antibiotics. In acute lung infection, treatment of mice with a single dose of azithromycin or doxycycline resulted in an inability to culture the organism compared to untreated controls. However, 77 and 25% of the culture-negative lungs were positive by PCR, respectively, and no differences in lung histopathology were noted (Malinverni et al., 1995b). This study suggests that infection was not eradicated and raises the question as to whether persistent infection was induced earlier in the course of infection as a result of treatment. In hyperlipidemic apoE-deficient mice, following either a single or three intranasal inoculations starting at 8 weeks of age, *C. pneumoniae* could be cultured from the aorta for 1–2 weeks after the first inoculation, although the aorta remained PCR positive up to 28 weeks of age. These results suggest that the organism can establish persistent infection of the aorta. This was also supported by immunohistochemical detection of the organism in foam cells in 24 week old mice (Moazed et al., 1997, 1999). Significantly, two independent studies were done in this model in which mice were infected and treated with azithromycin at a dose that is comparable to that given to humans for chlamydial respiratory infection (Rothstein et al., 2001; Blessing et al., 2005). In the first study, mice were infected twice, 1 week apart, and treated with azithromycin 2 weeks after each inoculation (Rothstein et al., 2001). In the other study, mice were infected mice three times, 1 week apart and received a 6 week course of azithromycin. In the latter study, mice were treated on days 3, 4, and 5 after the third infection and once a week for 5 subsequent weeks (Blessing et al., 2005). Neither treatment regimen had any beneficial effects on *C. pneumoniae* accelerated atherosclerosis. In the first study, at the endpoint of 26 weeks of age (12 weeks after the second inoculation), *C. pneumoniae* DNA was identified in lung, heart and aorta in 50% of both treated and untreated mice. An earlier study in New Zealand White rabbits treated with azithromycin for 7 weeks immediately following the third infection, demonstrated a decrease in *C. pneumoniae* accelerated intimal thickening. However, *C. pneumoniae* antigen was still detected in 3/10 treated rabbits in comparison to 2/9 untreated animals (Muhlestein et al., 1998). Fong et al. found that the time of treatment with antibiotic was key to mitigating the effect of *C. pneumoniae* infection on atherosclerosis development in rabbits. Early treatment of acute infection with clarithromycin, resulted in reduced effects; however, with delayed treatment there was not a statistically significant reduction in the detection of organism in atherosclerotic tissues in comparison to untreated rabbits. These studies provide further evidence that *C. pneumoniae* establishes persistent infection *in vivo*, which is refractory to antibiotic intervention.

A large number of studies from independent laboratories demonstrated the presence of the organism within human atherosclerotic tissue by detection of *C. pneumoniae* antigen and/or DNA (Campbell and Kuo, 2004; Taylor-Robinson and Boman, 2005). However, isolation of the organism has been rare (Ramirez, 1996; Jackson et al., 1997), suggesting that *C. pneumoniae* establishes persistent infection in the vasculature. Unfortunately, there are no clearly defined markers of persistent infection in humans. To identify such markers, Borel and her colleagues applied tissue microarray (TMA) technology coupled with immunohistochemistry using antibodies prepared against proteins that were differentially expressed *in vitro* in a gamma-interferon induced model of *C. pneumoniae* persistence (Molestina et al., 2002; Mukhopadhyay et al., 2006) and examined archived tissues from patients undergoing heart transplantation. An advantage of this tissue set was that *C. pneumoniae* had been detected in 7 of 12 patients by various methods and the organism was cultured from 1 of these patients (Ramirez, 1996). By TMA analysis, heart tissue from 10 of 12 patients were positively stained with antibodies against proteins upregulated in the persistent state (GroEL and GroES) and all were negative when stained with an antibody against a downregulated protein (Borel et al., 2006). Using a subset of these specimens, “aberrant” forms were visualized by transmission electron microscopy (TEM) and immunogold labeling with antibodies against GroEL and GroES. These forms were confirmed as *C. pneumoniae* by double labeling with other *C. pneumoniae* specific antibodies providing evidence of persistent infection in atheromas (Borel et al., 2008). A recent prospective study stained coronary heart tissue from patients undergoing heart transplants with a panel of antibodies against *C. pneumoniae* proteins upregulated in “aberrant” forms and detected antigen in 11 of 13 patients, supporting the notion that these antigens may serve as a marker of persistent infection (Borel et al., 2012). However, none of the tissues were PCR positive, nor was any ultrastructural evidence of the organism observed by TEM (Borel et al., 2012). The latter may reflect sampling size and the limited area of the atheroma that can reasonably be analyzed by this method. In our experience with immunohistochemical staining, detection of the organism in atherosclerotic lesions is localized and analysis of sequential sections can yield disparate results. The negative PCR results are more difficult to interpret when compared with the studies of archived tissue, although sampling may again play a role.

In conclusion, the negative outcome of the antibiotic trials should not result in dismissing substantive evidence supporting *C. pneumoniae* infection as a potential contributor to atherosclerotic processes without rigorous investigation of other factors that may alternatively explain the lack of benefits (or not). One of these is the ability of chlamydiae to establish persistent infection, a state that is refractory to antibiotic treatment. The availability of mouse models of persistent chlamydial infection should be exploited to specifically address whether: (1) antibiotics induce persistent *C. pneumoniae* infection in the vasculature; (2) persistent infection of the vasculature can be reactivated by immunosuppression; (3) the absence of an effect of antibiotic intervention on *C. pneumoniae* accelerated atherosclerosis is due to persistent infection and (4) transcriptional profiles that characterize persistence can be demonstrated *in vivo* as recently demonstrated for *C. muridarum* (Carey et al., 2013). More challenging is the identification of diagnostic markers or transcriptional signature patterns of persistent viable *C. pneumoniae* infection in humans, which may differ from those observed in experimental models of persistent infection and vary depending on the environmental factors in the host contributing to persistent infection in different anatomical sites.

## References

[B1] Anderson J. L. (2005). Infection, antibiotics, and atherothrombosis–end of the road or new beginnings? N. Engl. J. Med. 352, 1706–1709 10.1056/NEJMe058019 15843674

[B2] Beatty W. L. Morrison R. P. Byrne G. I. (1994). Persistent chlamydiae: from cell culture to a paradigm for chlamydial pathogenesis. Microbiol. Rev. 58, 686–699 7854252 10.1128/mr.58.4.686-699.1994 PMC372987

[B3] Blessing E. Campbell L. A. Rosenfeld M. E. Chesebro B. Kuo C. C. (2005). A 6 week course of azithromycin treatment has no beneficial effect on atherosclerotic lesion development in apolipoprotein E-deficient mice chronically infected with *Chlamydia pneumoniae*. J. Antimicrob. Chemother. 55, 1037–1040 10.1093/jac/dki128 15845780

[B4] Blessing E. Campbell L. A. Rosenfeld M. E. Chough N. Kuo C. C. (2001). *Chlamydia pneumoniae* infection accelerates hyperlipidemia induced atherosclerotic lesion development in C57BL/6J mice. Atherosclerosis 158, 13–17 10.1016/S0021-9150(00)00758-9 11500169

[B35] Borel N. Mukhopadhyay S. Kaiser C. Sullivan E. D. Miller R. D. Timms P. (2006). Tissue MicroArray (TMA) analysis of normal and persistent *Chlamydophila pneumoniae* infection. BMC Infect. Dis. 6:152 10.1186/1471-2334-6-152 17052347 PMC1622754

[B36] Borel N. Pospischil A. Dowling R. D. Dumrese C. Gaydos C. A. Bunk S. (2012). Antigens of persistent *Chlamydia pneumoniae* within coronary atheroma from patients undergoing heart transplantation. J. Clin. Pathol. 65, 171–177 10.1136/jclinpath-2011-200270 22049224

[B5] Borel N. Summersgill J. T. Mukhopadhyay S. Miller R. D. Ramirez J. A. Pospischil A. (2008). Evidence for persistent *Chlamydia pneumoniae* infection of human coronary atheromas. Atherosclerosis 199, 154–161 10.1016/j.atherosclerosis.2007.09.026 18028932

[B6] Campbell L. A. Kuo C. C. (2004). *Chlamydia pneumoniae*–an infectious risk factor for atherosclerosis? Nat. Rev. Microbiol. 2, 23–32 10.1038/nrmicro796 15035006

[B10] Cannon C. P. Braunwald E. McCabe C. H. Grayston J. T. Muhlestein B. Giugliano R. P. (2005). Antibiotic treatment of *Chlamydia pneumoniae* after acute coronary syndrome. N. Engl. J. Med. 352, 1646–1654 10.1056/NEJMoa043528 15843667

[B7] Carey A. J. Huston W. M. Cunningham K. A. Hafner L. M. Timms P. Beagley K. W. (2013). Characterization of *in vitro Chlamydia muridarum* persistence and utilization in an *in vivo* mouse model of Chlamydia vaccine. Am. J. Reprod. Immunol. 69, 475–485 10.1111/aji.12093 23414449

[B20] Carter J. D. Espinoza L. R. Inman R. D. Hudson A. P. (2010). Combination antibiotics as a treatment for chronic Chlamydia-induced reactive arthritis: a double-blind, placebo-controlled, prospective trial. Arthritis Rheum. 62, 1298–1307 10.1002/art.27394 20155838 PMC2907099

[B11] Danesh J. (2005). Antibiotics in the prevention of heart attacks. Lancet 365, 365–367 10.1016/S0140-6736(05)17842-8 15680439

[B12] Deniset J. F. Pierce G. N. (2010). Possibilities for therapeutic interventions in disrupting *Chlamydophila pneumoniae* involvement in atherosclerosis. Fundam. Clin. Pharmacol. 24, 607–617 10.1111/j.1472-8206.2010.00863.x 20653790

[B13] Epstein S. E. Zhu J. Najafi A. H. Burnett M. S. (2009). Insights into the role of infection in atherogenesis and in plaque rupture. Circulation 119, 3133–3141 10.1161/CIRCULATIONAHA.109.849455 19546396

[B14] Fong I. W. (2000). Antibiotics effects in a rabbit model of Chlamydia pneumoniae-induced atherosclerosis. J. Infect. Dis. 181(Suppl. 3), S514–S518 10.1086/315607 10839750

[B21] Gieffers J. Fullgraf H. Jahn J. Klinger M. Dalhoff K. Katus H. A. (2001). *Chlamydia pneumoniae* infection in circulating human monocytes is refractory to antibiotic treatment. Circulation 103, 351–356 10.1161/01.CIR.103.3.351 11157684

[B15] Gieffers J. Rupp J. Gebert A. Solbach W. Klinger M. (2004). First-choice antibiotics at subinhibitory concentrations induce persistence of *Chlamydia pneumoniae*. Antimicrob. Agents Chemother. 48, 1402–1405 10.1128/AAC.48.4.1402-1405.2004 15047553 PMC375316

[B16] Grayston J. T. (2003). Antibiotic treatment of atherosclerotic cardiovascular disease. Circulation 107, 1228–1230 10.1161/01.CIR.0000056032.56396.89 12628937

[B23] Grayston J. T. Kronmal R. A. Jackson L. A. Parisi A. F. Muhlestein J. B. Cohen J. D. (2005). Azithromycin for the secondary prevention of coronary events. N. Engl. J. Med. 352, 1637–1645 10.1056/NEJMoa043526 15843666

[B17] Hu H. Pierce G. N. Zhong G. (1999). The atherogenic effects of chlamydia are dependent on serum cholesterol and specific to *Chlamydia pneumoniae*. J. Clin. Invest. 103, 747–753 10.1172/JCI4582 10074493 PMC408120

[B18] Jackson L. A. Campbell L. A. Kuo C. C. Rodriguez D. I. Lee A. Grayston J. T. (1997). Isolation of *Chlamydia pneumoniae* from a carotid endarterectomy specimen. J. Infect. Dis. 176, 292–295 10.1086/517270 9207386

[B19] Jaff M. R. Dale R. A. Creager M. A. Lipicky R. J. Constant J. Campbell L. A. (2009). Anti-chlamydial antibiotic therapy for symptom improvement in peripheral artery disease: prospective evaluation of rifalazil effect on vascular symptoms of intermittent claudication and other endpoints in *Chlamydia pneumoniae* seropositive patients (PROVIDENCE-1). Circulation 119, 452–458 10.1161/CIRCULATIONAHA.108.815308 19139383

[B8] Jespersen C. M. Als-Nielsen B. Damgaard M. Hansen J. F. Hansen S. Helo O. H. (2006). Randomised placebo controlled multicentre trial to assess short term clarithromycin for patients with stable coronary heart disease: CLARICOR trial. BMJ 332, 22–27 10.1136/bmj.38666.653600.55 16339220 PMC1325128

[B22] Joensen J. B. Juul S. Henneberg E. Thomsen G. Ostergaard L. Lindholt J. S. (2008). Can long-term antibiotic treatment prevent progression of peripheral arterial occlusive disease? A large, randomized, double-blinded, placebo-controlled trial. Atherosclerosis 196, 937–942 10.1016/j.atherosclerosis.2007.02.025 17418218

[B24] Kutlin A. Roblin P. M. Hammerschlag M. R. (2002a). Effect of prolonged treatment with azithromycin, clarithromycin, or levofloxacin on Chlamydia pneumoniae in a continuous-infection Model. Antimicrob. Agents Chemother. 46, 409–412 10.1128/AAC.46.2.409-412.2002 11796350 PMC127037

[B25] Kutlin A. Roblin P. M. Hammerschlag M. R. (2002b). Effect of gemifloxacin on viability of *Chlamydia pneumoniae* (Chlamydophila pneumoniae) in an *in vitro* continuous infection model. J. Antimicrob. Chemother. 49, 763–767 10.1093/jac/dkf029 12003969

[B26] Laitinen K. Laurila A. L. Leinonen M. Saikku P. (1996). Reactivation of *Chlamydia pneumoniae* infection in mice by cortisone treatment. Infect. Immun. 64, 1488–1490 8606126 10.1128/iai.64.4.1488-1490.1996 PMC173951

[B27] Malinverni R. Kuo C. C. Campbell L. A. Grayston J. T. (1995a). Reactivation of *Chlamydia pneumoniae* lung infection in mice by cortisone. J. Infect. Dis. 172, 593–594 10.1093/infdis/172.2.593 7622914

[B28] Malinverni R. Kuo C. C. Campbell L. A. Lee A. Grayston J. T. (1995b). Effects of two antibiotic regimens on course and persistence of experimental *Chlamydia pneumoniae* TWAR pneumonitis. Antimicrob. Agents Chemother. 39, 45–49 10.1128/AAC.39.1.45 7695327 PMC162482

[B29] Moazed T. C. Campbell L. A. Rosenfeld M. E. Grayston J. T. Kuo C. C. (1999). *Chlamydia pneumoniae* infection accelerates the progression of atherosclerosis in apolipoprotein E-deficient mice. J. Infect. Dis. 180, 238–241 10.1086/314855 10353889

[B30] Moazed T. C. Kuo C. Grayston J. T. Campbell L. A. (1997). Murine models of *Chlamydia pneumoniae* infection and atherosclerosis. J. Infect. Dis. 175, 883–890 10.1086/513986 9086145

[B31] Molestina R. E. Klein J. B. Miller R. D. Pierce W. H. Ramirez J. A. Summersgill J. T. (2002). Proteomic analysis of differentially expressed *Chlamydia pneumoniae* genes during persistent infection of HEp-2 cells. Infect. Immun. 70, 2976–2981 10.1128/IAI.70.6.2976-2981.2002 12010987 PMC127979

[B32] Muhlestein J. B. (2011). Chronic infection and coronary atherosclerosis. Will the hypothesis ever really pan out? J. Am. Coll. Cardiol. 58, 2007–2009 10.1016/j.jacc.2011.08.015 22032714

[B33] Muhlestein J. B. Anderson J. L. Hammond E. H. Zhao L. Trehan S. Schwobe E. P. (1998). Infection with *Chlamydia pneumoniae* accelerates the development of atherosclerosis and treatment with azithromycin prevents it in a rabbit model. Circulation 97, 633–636 10.1161/01.CIR.97.7.633 9495296

[B34] Mukhopadhyay S. Miller R. D. Sullivan E. D. Theodoropoulos C. Mathews S. A. Timms P. (2006). Protein expression profiles of *Chlamydia pneumoniae* in models of persistence versus those of heat shock stress response. Infect. Immun. 74, 3853–3863 10.1128/IAI.02104-05 16790757 PMC1489704

[B9] O'Connor C. M. Dunne M. W. Pfeffer M. A. Muhlestein J. B. Yao L. Gupta S. (2003). Azithromycin for the secondary prevention of coronary heart disease events: the WIZARD study: a randomized controlled trial. JAMA 290, 1459–1466 10.1001/jama.290.11.1459 13129985

[B37] Phillips Campbell R. Kintner J. Whittimore J. Schoborg R. V. (2012). *Chlamydia muridarum* enters a viable but non-infectious state in amoxicillin-treated BALB/c mice. Microbes Infect. 14, 1177–1185 10.1016/j.micinf.2012.07.017 22943883 PMC3654801

[B38] Ramirez J. A. (1996). Isolation of Chlamydia pneumoniae from the coronary artery of a patient with coronary atherosclerosis. The *Chlamydia pneumoniae*/Atherosclerosis Study Groupgroup. Ann. Intern. Med. 125, 979–982 10.7326/0003-4819-125-12-199612150-00008 8967709

[B39] Rosenfeld M. E. Campbell L. A. (2011). Pathogens and atherosclerosis: update on the potential contribution of multiple infectious organisms to the pathogenesis of atherosclerosis. Thromb. Haemost. 106, 858–867 10.1160/TH11-06-0392 22012133

[B40] Rothstein N. M. Quinn T. C. Madico G. Gaydos C. A. Lowenstein C. J. (2001). Effect of azithromycin on murine arteriosclerosis exacerbated by *Chlamydia pneumoniae*. J. Infect. Dis. 183, 232–238 10.1086/317941 11120929

[B41] Taylor-Robinson D. Boman J. (2005). The failure of antibiotics to prevent heart attacks. BMJ 331, 361–362 10.1136/bmj.331.7513.361 16096289 PMC1184236

[B42] Wyrick P. B. Knight S. T. (2004). Pre-exposure of infected human endometrial epithelial cells to penicillin *in vitro* renders *Chlamydia trachomatis* refractory to azithromycin. J. Antimicrob. Chemother. 54, 79–85 10.1093/jac/dkh283 15163653

